# Preserved motor learning after stroke is related to the degree of proprioceptive deficit

**DOI:** 10.1186/1744-9081-5-36

**Published:** 2009-08-28

**Authors:** Eric D Vidoni, Lara A Boyd

**Affiliations:** 1 Department of Neurology, University of Kansas Medical Center, Kansas City, USA; 2 Department of Physical Therapy, University of British Columbia Vancouver, British Columbia, Canada

## Abstract

**Background:**

Most motor learning theories posit that proprioceptive sensation serves an important role in acquiring and performing movement patterns. However, we recently demonstrated that experimental disruption of proprioception peripherally altered motor performance but not motor learning in humans. Little work has considered humans with central nervous system damage. The purpose of the present study was to specifically consider the relationship between proprioception and motor learning at the level of the central nervous system in humans.

**Methods:**

Individuals with chronic (> 6mo) stroke and similarly aged healthy participants performed a continuous tracking task with an embedded repeating segment over two days and returned on a third day for retention testing. A limb-position matching task was used to quantify proprioception.

**Results:**

Individuals with chronic stroke demonstrated the ability to learn to track a repeating segment; however, the magnitude of behavioral change associated with repeated segment-specific learning was directly related to the integrity of central proprioceptive processing as indexed by our limb-position matching task.

**Conclusion:**

These results support the importance of central sensory processing for motor learning. The confirmation of central sensory processing dependent motor learning in humans is discussed in the context of our prior report of preserved motor learning when sensation is disrupted peripherally.

## Background

It is commonly held that sensory feedback plays an important role in motor skill learning [1-3]. Physiologic evidence from animal lesion models suggests that sensory cortex is necessary for learning new skills, including movement sequences. For example, lesions to primary somatosensory cortex (S1) in cats [4] and non-human primates [5] inhibit motor learning. Animals with experimentally induced lesions to S1 fail to acquire new motor skills with the contralesional hand. However, once a skill has been acquired, discrete S1 lesions do not significantly interfere with existing skill performance [5]. Though activation of the sensory cortex has also been linked to excitation of the human motor cortex [6], and disruption of parietal activity interferes with adaptation [7] little other direct evidence links somatosensory processing to motor learning in humans.

Further clouding our understanding of the interaction of sensory and motor systems are reports that somatosensation is not essential to motor adaptation. Several studies have examined motor performance and adaptation in individuals lacking proprioception [8-13]. Taken together, these studies have noted maintenance of adaptation and control. Indeed, we recently demonstrated that peripherally altering proprioception in healthy individuals did not adversely affect motor sequence learning [14]. To disrupt proprioception peripherally, participants' arms were vibrated continuously while they practiced a repeated pattern of continuous movements. We discovered that participant performance was compromised by vibration during practice; however, at retention when vibration was removed, individuals performed the repeated portion of the movement as well as controls. Our findings supported previous work and suggested that when peripheral proprioception is disrupted other sensory systems can compensate for altered sensation and provide the feedback that is necessary for motor learning.

One apparent difference between our peripheral disruption of proprioception and the animal lesion models is continuity of the central somatosensory processing system. Preservation of the central nervous system allows for comparison and weighting of incoming, sensory information so that skill learning is maintained, even when feedback sources are conflicting or aberrant. Conversely, when the central structures are damaged, feedback evaluation, sensory reception, interpretation or expectation may be diminished, disrupting motor learning [15,16].

The present study was designed as a follow-up investigation, to test whether central proprioceptive processing dysfunction impacts continuous motor learning. It further represents a corollary study in humans to animal work that demonstrated the importance of central sensory processing for motor learning [4,5,17]. Continuous sequencing tasks allow for the study of emergent procedural learning [18]; because of the need for constant updating, continuous tasks are highly reliant on the proprioceptive system especially in the absence of visual feedback. This is in contrast to other commonly employed motor learning paradigms that employ discrete end-point movements, which can be largely planned in advance [19]. Continuous tracking also differs from studies requiring a reach to discrete targets in a novel environment [20], where a commonly performed behavior is adapted to new dynamics. The task employed here requires extended and accurate gross motor control of the upper extremity in coordinated patterns of movement. Finally, in the present study we considered the pattern of continuous velocity changes rather than absolute position [18,21,22] as recent work has emphasized the encoding of velocity-based information by the proprioceptive system [23,24].

We hypothesized that if central processing of proprioceptive feedback were necessary for motor learning, stroke-related damage to somatosensory cortical areas, thalamus and/or the associated white matter tracts would result in impaired continuous motor learning. Furthermore, we expected that the magnitude of learning-related change in motor behavior would be related to the degree of proprioceptive impairment as measured by limb-position matching ability. We tested these hypotheses using a continuous tracking task in which we eliminated useable visual feedback. In addition, we performed a separate retention test to dissociate motor learning from transient performance-related improvements [25].

## Methods

### Participants

Twelve individuals presenting with clinical signs of a chronic stroke (at least 6 months post) [26,27] in the middle cerebral artery distribution (CVA group), and 9 similarly aged individuals neurologically intact individuals (Healthy Control; HC group) were recruited from the greater Vancouver, British Columbia and Kansas City, Kansas communities and provided informed consent to participate in this study. The study was approved by the Institutional Review Boards of the University of Kansas Medical Center and the University of British Columbia. All participants had near vision corrected to at least 20/40, and no history of diabetes or peripheral neurological damage. Prospective participants were screened using the Mini-Mental State Exam (MMSE) [28]. Decreased motor learning capacity and proprioception occurs with advancing age [29-32]. Therefore it was critical that the age of our HC group share a similar age distribution as our participants with stroke. Group characteristics are summarized in Table 1. Data from 2 CVA participants were excluded from analysis secondary to consistently poor tracking as defined in the methods section (resulting CVA n = 10). The upper extremity motor portion of the Fugl-Meyer stroke recovery assessment (UEFM) provides a measure of motor function and was administered to all participants in the CVA group [33]. Individuals were compensated for travel expenses associated with participation in this research.

**Table 1:** Participant characteristics

	Age	MMSE	LPM	UEFM	Lesion
CVA1	70	30	1.34	50	L Corona Radiata & Putamen
CVA2	67	29	1.16	59	L PLIC & Thalamus
CVA3	75	29	2.03	51	R Corona Radiata & Putamen
CVA4	45	28	0.91	60	L Occip. lobe, Parahippocampal g & Thalamus
CVA5	60	25	1.55	54	R Temporal, Parietal, Insular & Occipital lobes
CVA6	65	28	1.18	51	R Corona Radiata, PLIC & Thalamus
CVA7	73	29	1.36	32	R Caudate, Corona Radiata, Putamen & PLIC
CVA8	32	28	2.00	25	*L Middle cerebral artery distribution*
CVA9	62	29	2.17	24	*Unavailable*
CVA10	66	29	0.64	56	*R Inferior Parietal & Temporal lobes*

Avg. (SD)	61.5 (13.3)	28.4 (1.3)	1.52 (0.4)	46.2 (13.8)	

HC1	61	29	0.93	--	--
HC2	62	30	1.22	--	--
HC3	59	30	0.74	--	--
HC4	30	30	1.27	--	--
HC5	66	30	0.91	--	--
HC6	73	29	0.98	--	--
HC7	63	30	0.71	--	--
HC8	46	30	0.77	--	--
HC9	63	30	1.48	--	--

Avg. (SD)	58.1 (12.7)	29.8 (0.4)	1.0 (0.3)	--	--

Individual characteristics and group averages (SD) including Age, Mini-Mental State Exam (MMSE) score, Upper Extremity Fugl-Meyer (UEFM) score and normalized Limb Position Matching (LPM) index. Stroke location is also presented (PLIC = internal capsule, posterior limb). Location of the stroke based on medical records for individuals unable to complete an MRI are presented in italics. CVA9 presented with clinical signs of a right middle cerebral artery infarct, including L hemiparesis and increased muscle tone.

### Lesion location

Neuroanatomical scans were performed on 7 of the 10 CVA participants included in analysis and MRI reports were obtained for 2 of the remaining 3 individuals. The 3 individuals for whom scans were not obtained were ineligible for MR imaging at the time of the study. Participant-specific lesion information is reported in Table 1.

### Task

**Figure 1:**
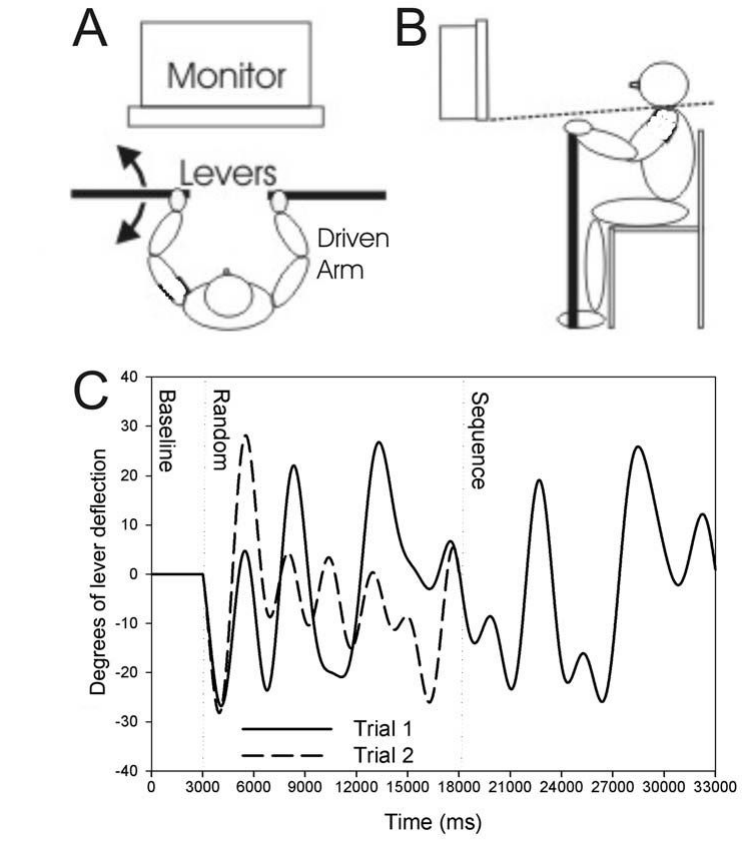
**Tracking task**. A) Participants were seated before a computer monitor and griped one (tracking task) or both (limb position matching task) horizontally mounted levers. Draping is drawn over the shoulders to prevent visualization of arm movement, represented by a dashed line in B. C) All participants tracked a target following movement patterns similar to these two example trials. Following a 3s stable baseline, a sine-cosine waveforms dictated target movement in degrees up or down at the horizontal center of the screen. Two full trial waveform patterns, each consisting of 1 random and 1 repeated segment, are overlaid in the diagram to demonstrate that all trials had an identical segment common to each. The random segment comes first, followed by the repeated segment during both trials for ease of visualization.

The continuous tracking task [34] used in the present study was similar to that reported previously [14,35]. Participants were seated before a computer monitor and asked to grasp a horizontally mounted lever restricted to movement in the transverse plane. A push/pull, shoulder/elbow flexion and extension motion was used to control an on-screen cursor (Figure 1A &1B). Either the dominant arm (HC) as determined by the Edinburgh Inventory [36] or the hemiparetic arm (CVA) was used to manipulate the lever. Participants were instructed to track the target moving vertically at the midline of the screen as accurately as possible by controlling the cursor with the lever. Target and lever position were sampled at 40 Hz using custom software developed on the LabView platform (v. 7.1; National Instruments, Austin, TX). Lever excursion of 60°, equal to an arc length of 31 cm, was required to accurately track the target. One participant with stroke could not achieve this range of motion. For this participant, the experimental software was calibrated such that the maximum excursion necessary to perform the task was ~2 cm less than the participant's achievable excursion. In addition, an elastic wrap was used to maintain the hand position of two individuals with stroke on the lever due to weak grip strength and/or poor sensation.

To maximize dependence on proprioceptive information, we used our previously established protocol to severely restrict visual feedback of movements [14]. Draping was placed over but not in contact with the participant's upper body to prevent vision of the arms. Additionally, over the first 20 practice trials, visual feedback that had been provided to aid participants' initial understanding of the task was faded [14]. To accomplish this on day 1, arm position information (i.e. cursor) was linearly faded from continuous presentation on trial 1, to only a 200 ms duration presentation every 2s by trial 19. Visual feedback was maintained at this frequency for the rest of the experiment. This frequency of position feedback is *well below *that which Kao [37] reported to be virtually useless for guiding continuous hand-controlled cursor movements. To encourage and motivate individuals for this difficult task, a single summary feedback score regarding overall tracking accuracy was provided after each trial, as a percentage of time the position cursor spent within a 10° bandwidth of the target.

### Procedure

Individuals practiced 50 trials of continuous tracking on each of two days. Each session lasted approximately 1 hour. On a separate third day, participants returned for 10 retention test trials. These trials required the same tracking activity as practiced on the 2 training days. No practice trials were allowed prior to testing on any day.

The pattern of target movement was a predefined sine-cosine waveform constructed according to a method modified from Wulf and Schmidt [21]. In continuous tracking paradigms, a segment of the tracked waveform is often embedded regularly, such that the participant receives repeated practice on the same set of movements. Through this repeated regular practice the subject may learn aspects of the segment motion. Performance on the repeated segment can be compared to performance on random, novel segments, i.e. those that have never been practiced before, to index segment-specific learning. For this study a unique 33s trial was seamlessly constructed from one 3s baseline and two 15s sine-cosine waveforms, or segments. During each trial, participants attempted to track one novel random segment and one repeated segment that was identical in every trial (Figure 1C).

The use of a random and repeated segment is important for two reasons. First, improved tracking can come from two sources: general improvement and understanding of task dynamics, or improvement on a specific pattern of movement. Repeated segment-specific learning involves acquisition and retention of the precise pattern of a practiced movement, for example, signing your name. In contrast, general task learning is improvement in the non-specific components of the task. In the signature example, this would include learning the necessary hand force to grip a pen. In continuous tracking experimental paradigms, improvement on the random segment is considered to be an indication of general task learning [21]. This can include familiarity with the apparatus or requirement of the task among other factors. Improvement on the repeated segment is considered to reflect pattern specific motor learning of the repeated segment beyond general task learning [21,26]. The difference between improvement on the repeated and random segments indexes the improvement that is solely related to practice of the repeated motor pattern. Further, comparison of the random and repeated segment tracking for each individual controls for performance differences between subjects such as those based on skill level or handedness.

Individuals were never informed of the existence of this repeated segment. The presentation of this repeated segment, first or last in a trial, was randomized to minimize order effects. However, the same overall trial order was employed for every participant. That is, each participant practiced the same trials in the same order throughout the study to allow for comparison.

### Indexing of proprioception

The ability to access and discriminate proprioceptive information was indexed via a common clinical assessment, limb position matching [38]; In a proprioceptive assessment for stroke, the hemiparetic limb is moved by a clinician while the patient attempts to match those movements with the non-hemiparetic limb. We modified this assessment for more precise, numeric quantification of altered proprioception and have previously demonstrated its sensitivity [14]. Two near-frictionless, horizontally-mounted levers, the same used in training, were grasped in each hand (Figure 1A). For individuals in the stroke group, the experimenter supported the more involved, hemiparetic arm at the humeral condyles with minimal cutaneous contact and drove the lever through a 30 second continuous pattern of random movements. Participants closed their eyes and matched the movement of the driven arm by moving the opposite, less involved, non-hemiparetic arm. The same procedure was followed for those in the healthy control group with the dominant arm being driven by the experimenter. Limb position was smoothed using a 100 ms moving average [39] to reduce noise and corrected for constant error. As has been previously reported, the area difference between the position of the driven and matching arms, root-mean-squared error (RMSE, see Appendix 1), was then calculated [40]. To generate a limb position matching score (LPM) that would be comparable across groups, inter-limb matching error for each CVA participant was normalized to the average inter-limb matching error from the healthy control group (see Appendix 2). In this calculation, 1.0 indicates average inter-limb position matching; greater values reflect progressively worse matching.

### Outcome measures

For our experimental continuous motor learning task, target and lever position data were differentiated into the velocity profile and smoothed using a 100 ms moving average. We considered the pattern of continuous velocity changes rather than absolute position [18,21,22] as recent work has emphasized the encoding of velocity-based information by the proprioceptive system [14,23,24]. Correlation between target and arm velocity profiles was performed for each trial. Trials were excluded from analysis if the correlation coefficient did not reach r = 0.3. As presented earlier, two participants were removed from analysis, having tracked poorly in more than 50% of trials based on this criterion (total CVA n = 10). Anecdotally, these individuals had great difficult with even basic movements. For the remaining participants, less than 3% of trials were excluded. RMSE between target and arm velocity profiles was calculated separately for random and repeated segments and was averaged across sets of 10 consecutive trials to group data into blocks (each 10 trials = 1 block of data). This procedure was repeated for all trials across the two practice days and at retention.

To quantify repeated segment-specific learning we separately calculated the improvement in average RMSE for random and repeated segments. The difference between improvement on the random and repeated segment RMSE from Block 1 to Retention was defined as the repeated segment-specific improvement score (SSI). Because this change score is calculated separately for each individual it normalizes for baseline differences between groups as well as other inter-subject differences that might be related to hand dominance, motor experience, or stroke severity.

### Statistical analyses

First, to examine initial tracking performance and segment differences, an omnibus three-way ANOVA of Group (CVA, HC), Segment (random, repeated) and Block (acquisition 1 through 10, and retention) RMSE with repeated measures correction of Segment and Block was conducted. The ANOVA was tested at α = 0.05 and Greenhouse-Geisser correction was employed. To further examine repeated segment-specific learning we then performed separately, at Block 1 and Retention, planned two-way ANOVAs (Segment by Group) with repeated measures correction of Segment. The planned, post-hoc ANOVAs afford the ability to compare initial and final performance between those with and without stroke at the beginning and the end of the study and to confirm the skill was learned.

To examine the relationship between repeated segment-specific learning and sensorimotor indices, correlations were performed between LPM or UEFM and SSI. We additionally performed partial correlations to control for age effects on proprioception. The two-way ANOVAs and correlations were tested at α = 0.025 to correct for multiple comparisons.

## Results

### Tracking accuracy

Individuals with stroke were generally less accurate at tracking throughout the study. The three-way ANOVA a result confirmed this observation with significant a main effect of Group (F(1,17) = 8.76, p = 0.009). Our planned comparison of Block 1 revealed no initial Group difference in tracking RMSE (p = 0.129). However, by Retention the HC group tracked with significantly less error (F(1,17) = 10.39, p = 0.005). No interaction effect with group was detected for any ANOVA.

**Figure 2:**
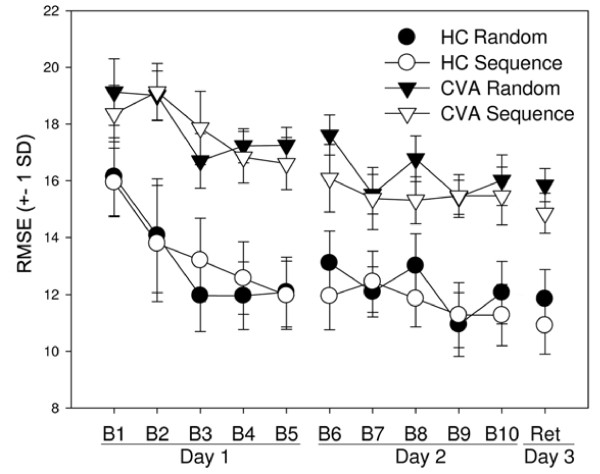
**Task performance and learning**. RMSE (+- 1 SD) of velocity over the course of training and retention testing for CVA (triangles) and HC (circles) participants. Throughout the study, the HC group was more accurate. At the beginning of practice, Block 1, there is no difference between random (black) and repeated (white) segments. Over the course of training both groups improved on the repeated segment to a greater extent than the random segment. This improvement persisted at retention testing with both groups exhibiting repeated segment-specific learning.

Despite overall difference in tracking accuracy, both groups demonstrated repeated segment-specific learning over the course of the experiment. This was confirmed via a three-way ANOVA which yielded a significant interaction of Block and Segment (F(10,170) = 8.14, p = 0.011). Visual inspection indicates this effect is a function of greater improvement over practice on the repeated segment (Figure 2) as compared with the random segment. This was confirmed with our two-way ANOVA analysis. RMSE between segments in Block 1 were not different, indicating similar performance (p = 0.053). However, at Retention both groups had lower RMSE on the repeated segment than the random segment demonstrating repeated segment-specific learning (F(1,17) = 29.91, p < 0.001).

### Relationship between learning and proprioception

**Figure 3:**
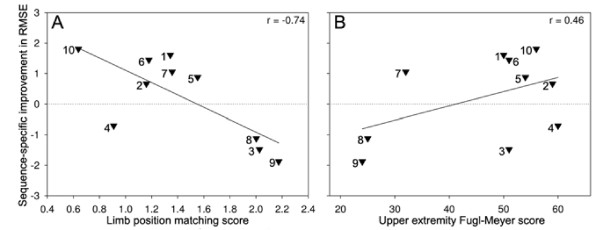
**Proprioception, motor control and repeated segment-specific learning**. Proprioceptive processing ability indexed as the normalized limb position matching RMSE (LPM) is plotted against the segmental difference in repeated segment-specific improvement over the course of practice (A). LPM values greater than 1.0 represent worse matching error than the average HC participant. Increasing values of repeated segment-specific improvement (SSI) represent greater improvement on the repeated segment as compared to the random segment. A strong relationship was detected between LPM and repeated segment learning (r = -0.74, *p = 0.015). B) Motor function of the hemiparetic arm as indexed by the Upper Extremity Fugl-Meyer assessment (UEFM) is plotted against the segmental difference in repeated segment-specific improvement over the course of practice. A moderate but non-significant (r = 0.46, p = 0.117) relationship between motor function and repeated segment-specific improvement (SSI) was detected. Increasing UEFM values represent better motor function. In both panels the horizontal dotted line represents general task improvement over practice; values above the line indicate repeated segment-specific learning. CVA group numbers corresponding to participants as identified in Table 1 are placed alongside each datapoint.

As expected, the CVA group demonstrated worse normalized limb position matching ability (LPM, average RMSE = 1.52 ± 0.4) than did the HC group (1.0 ± 0.3) (p = 0.017, one tailed t-test). The importance of proprioceptive processing accuracy for motor learning was illustrated by a strong [41] and significant relationship, between LPM and sequence-specific learning (SSI), (r = -0.74, p = 0.015; Figure 3A). Controlling for age using partial correlation made only nominal change (r = -0.76, p = 0.019). The relationship between general motor function (UEFM) and repeated segment-specific learning (SSI) was not significant prior to (r = 0.46, p = 0.117; Figure 3B) or after controlling for age (r = 0.41, p = 0.279). Because plotting of partial correlations involves difficult-to-interpret residual error, Figures 3A and 3B plot the simple relationship between primary measures, not accounting for age.

## Discussion

We demonstrated that following stroke, some individuals can learn a specific, repeating pattern of movement as a part of a continuous tracking task where minimal visual feedback is present. However, we also discovered that after the central nervous system (CNS) is damaged, proprioceptive integrity is closely related to the amount of behavioral change associated with repeated segment-specific learning. In contrast, general motor function demonstrated during random segment tracking is not strongly related to motor learning. Our findings support and extend previous research implicating proprioception in motor learning to include repeated segment-specific continuous tracking [42-44]. Importantly, the present study is among the first to examine the impact of damage to central proprioceptive processing ability on continuous motor learning.

### Motor learning task

At the beginning of practice both groups performed equally well on the random and repeated segments with sufficient visual feedback, suggesting that significant differences at retention were not the result of any baseline effects. Over the course of practice, a differential ability to improve on the random versus repeated segments of our tracking task became apparent. Prior tracking studies consistently report progressive spatial tracking improvement across practice, specifically on the repeated segment of the skill [21,22,34]. The findings presented here are consistent with this past work despite our use of velocity profiles rather than spatial movements as our dependent measure. Both groups in the present study were able to improve their general tracking ability and showed better performance for the specific, repeated portion of movement. Importantly, both groups improved on the repeated segment of the tracking task to a greater extent than the random portion with practice and thus show repeated segment-specific motor learning.

Because a random tracking segment was embedded within each trial, including the retention tests, we were able to dissociate effects of altered limb position sense on general task performance from the development of a learned plan for movement. Indeed, both groups demonstrated significant improvement on the repeated segment as compared to the random segment at retention, supporting previous findings of preserved motor learning capacity following stroke [26,45-47]. Often, work investigating the role of proprioception for motor performance has only considered single time points [42,43,48-50]. If we had employed such a design we may have not noted a relationship between motor learning related change and proprioceptive processing ability. However, because we examined multiple days of practice, and employed a delayed retention test, we were able to discover that the magnitude of learning related change was directly related to the preservation of proprioception as indexed via our limb matching task. To our knowledge, no prior studies regarding the role of proporioception in continuous motor learning and/or proprioception following stroke have employed a separate retention test design. Therefore, it has not been clear whether altered central processing of proprioceptive information would deleteriously impact motor learning. Taken together, these data indicate that the individuals with stroke who had poor limb position sense were at a disadvantage during learning of a continuous motor pattern as compared to those with intact position sense.

### Proprioception and motor learning

The present study supplements previous reports of altered novel skill learning following induced lesions to the sensory cortex in animal models [4,5]. We found that proprioception was strongly related to the magnitude of behavioral change associated with learning to accurately track a repeated pattern of movement, even after accounting for age. Because our measure of repeated segment-specific learning (SSI) reflects improvement beyond general, non-specific task learning, this finding argues against the suggestion that proprioception is merely important for changes in general motor control. Rather, it appears that proprioception was crucial for improved tracking accuracy specific to the practiced, *repeating pattern *of movement. This is consistent with previous suggestions that proprioception may be important for forming and helping to update a template of appropriate velocity-based motor commands for successful execution of a motor skill [2,23].

The relationship between proprioception and motor skill learning stands in contrast to our findings concerning arm motor function. Following stroke, arm motor function as indexed by the Fugl-Meyer scale did not correlate with the ability to improve tracking of repeated movement patterns. Because in the past, many studies of motor sequence learning following stroke required the use of the ipsilesional, less involved upper extremity [18,45,46], this relationship had previously been poorly characterized.

It may be argued that vision could have been used to supplement or compensate for proprioceptive deficit. Indeed, vision and proprioception have received considerable attention in the literature regarding their role during motor learning [49]. And it has been previously noted that vision is critical when proprioceptive sensation is diminished or absent [8,9]. To explore the contribution of proprioception without the confound of visual feedback, we reduced visual information available to the participant via several controls. First, we occluded vision of the arm via draping. Next, we quickly faded feedback regarding cursor position over the first 20 trials to an intermittency exceeding that which Kao [37] cited as being disruptive to continuous tracking. Finally, no vision was used in our proprioceptive measure, the limb position matching task (LPM). However, we chose to preserve some visual feedback to reduce cumulative error which might have obscured improved motor control associated with learning [51] by displaying the arm position cursor for 200 ms at 1800 ms intervals. It is possible that even this minimal visual information may have allowed participants to evaluate their performance and adjust accordingly in the absence of trustworthy proprioceptive feedback. However, based on the past work of Kao [27] and our own previous study [14] we find this explanation of our conclusions improbable.

### Peripheral vs. central disruption of proprioception

Our present finding that proprioceptive deficit is associated with capacity for change during motor learning initially appears at odds with our previous report that disruption of proprioception did not interfere with motor learning. However, we suggest that this difference is reconciled when the location of damage and integrity of the central nervous system is considered.

In our prior report [14] we found that disrupting proprioception with vibration interfered with performance, perhaps by masking proprioceptive signals used by the central nervous system to coordinate movements. However, this ultimately did not prevent participants from learning a specific set of movements. Several studies have examined motor performance and adaptation in individuals with large fiber neuropathy resulting in absent proprioception [8,9,13,49]. Taken together these studies have noted that skill learning is possible following deafferentation, though movements are clearly disrupted. Maintenance of the ability to learn new movements has also been reported after peripheral lesions such as in dorsal rhizotomies in non-human primates [52,53].

In contrast, the present finding that central processing of proprioceptive information is related to learning supports prior work in animals [5,6,17,54]. We suggest that this dichotomy is a function of the integrity of the sensory processing system, specifically somatosensory cortical areas, thalamus and the associated white matter tracts. Parietal cortex is known to maintain representations of the body [55]. When these sensory-associated regions are disrupted, by insult or by transient disturbance such as TMS [7] learning is compromised. If these structures remain intact, they are available to create representations, of behavior through intra-cortical interaction even when one or more sources of feedback are not dependable. Therefore it is not clear that the study of individuals with peripheral neuropathy or temporarily disrupted proprioception adequately addresses the role of central proprioceptive processing in motor skill learning.

## Conclusion

We recruited individuals with chronic stroke in the middle cerebral artery distribution, and similarly aged healthy controls to perform a continuous motor learning task. We severely restricted visual feedback and in this manner were able to examine the role of proprioception in motor learning. Despite the presence of a stroke, some individuals were able to demonstrate behavioral change and thus show learning of the practiced pattern of continuous movements. However, the degree of proprioceptive deficit was strongly related to the amount of change made. It should be noted that the size of the cohort and heterogeneity of the lesions warrant care when interpreting these findings. However, the findings support prior animal work implicating central proprioceptive capability as important for learning patterns of movement.

## Appendices

### Appendix 1

RMSE = SQRT(∑(xi - Xi)^2^/n) where xi = driven arm position (or target velocity during sequence training) and Xi = matching arm position (or tracking arm velocity during sequence training).

### Appendix 2

LPM = (Σx_i_/n)/Σ (Σy_i_/n)/r where x_i _= limb matching trial RMSE for CVA group, y_i _= limb matching trial RMSE for HC group, n = # of limb matching trials, r = # of HC participants.

## Competing interests

Neither author has any personal or financial relationship to declare regarding this manuscript.

## Authors' contributions

EDV participated in all aspects of the study. LAB participated in study design, interpretation of findings and drafting the manuscript. Both authors have read and approved the final version of this manuscript.
